# Survival rate and need for maintenance of cover-denture prostheses borne on three or less abutment teeth: ten-year results

**DOI:** 10.1186/s13005-026-00619-9

**Published:** 2026-04-21

**Authors:** Laurentia Schuster, Ali-Reza Ketabi, Till Dammaschke, Hans-Christoph Lauer

**Affiliations:** 1 Department of Periodontology and Operative Dentistry, University of Münster, Waldeyerstraße 30, 48149, Münster, Germany; 2 Department of Prosthodontics, School of Dentistry, Faculty of Health, Witten/Herdecke University, Witten, Germany; 3 Department of Prosthodontics, Johann Wolfgang Goethe-University Frankfurt, Frankfurt, Germany

**Keywords:** Overdenture prosthesis, Telescopic crown, Resilient telescopic crowns, Cover-denture prosthesis

## Abstract

**Statement of problem:**

When providing patients with cover-denture prostheses, even teeth with poor long-term prognosis can serve as abutment teeth. This has a positive psychological effect on patients and makes it easier to adapt them later to a complete denture.

**Purpose:**

Aim of the present study was to investigate the survival rate and need for maintenance of cover-denture prostheses.

**Methods:**

Data were collected from patient records. Statistical analysis was performed using Kaplan-Meier survival analyses, log-rank tests (*P* < 0.05), Cox regressions (*P* < 0.05), and Non-Parametric Mean Cumulative Function.

**Results:**

Two hundred twenty-seven cover-dentures in 204 patients were examined. Mean age was 67 years, mean follow-up time was 3.4 years. 27.75% of prostheses were borne on one abutment tooth, 40.97% on two and 31.28% on three abutment teeth. In 60.4% percent of cases, copings were cast from a precious alloy. The 5-year survival rate for cover-dentures was 64.5%. The most common complication (36.6%) was decementation of a coping. In 30.9% of cases, one or more abutment teeth fractured and in 28.7% of cases one or more abutment teeth were lost. Most common need for maintenance was the removal of pressure points in the prosthesis. In 29.5% of cases, a relining of the cover-dentures was necessary.

**Conclusion:**

A cover-denture prosthesis is a suitable therapeutical option for patients with few remaining teeth. The survival rate is comparable to that of other double crown retained removable partial dentures and occurring complications are manageable. Cover-denture prostheses represent an adequate alternative to complete extraction and can be recommended in clinical practice.

## Introduction

Around half of the adult population in Europe wears dentures [1]. Depending on tooth loss and the resulting change in chewing habits [2], a prosthetic restoration improves masticatory function and patient related parameters [3]. Decisive factors for the success of a prosthetic restoration include functional stability, high wearing comfort [4] and good distribution of the functional load between the alveolar ridge and abutment teeth [5]. Patients wearing a prosthetic restoration have a higher oral-health related quality of life than patients not having prosthetic restoration [6]. According to the *Glossary of Prosthodontic terms *[7] a prosthetic restoration is any type of material that aims to replace lost tooth structure, teeth and oral structures. Treatment options vary by gap size and distribution, and remaining teeth condition [8]. If implant placement is not feasible to augment the number of abutment teeth, removable partial dentures (RPDs) are a successful option. The choice of anchoring elements depends on the abutment teeth, aesthetics, and financial factors [9]. Suitability as abutment teeth is evaluated by endodontic, periodontal, and restorative criteria[10]. Older patients often have teeth with uncertain prognoses, requiring treatment plans that accommodate further tooth loss [11]. RPDs can increase caries risk and the extent of periodontal damage to the abutment teeth [12–14], which are the most common causes of abutment tooth loss [15]. Regular follow-up is essential to maintaining prosthetic function and periodontal health [14, 16].

If there are three or fewer possibly periodontally damaged teeth left, they may not be able to withstand the load of clasp- or double-crown-retained RPDs. Given its similarity to a full denture, a cover-denture prosthesis (CDP) is a proven alternative [15, 17–19]. A CDP resembles a full denture but is anchored to up to three abutment teeth with resilient telescopic crowns, which counteract lateral shear forces. These cylindrical crowns allow for controlled movement [17, 18], distributing force to the denture base first. A CDP helps prevent total edentulism after extractions and aids adaptation to a purely mucosa-supported full denture, as even teeth with periodontal damage can still be integrated as abutments [17]. Resiliently retained RPDs show good clinical survival, with abutment survival rates comparable to retentively retained RPDs, when resilient anchorage principles are followed [20]. Nevertheless, the indication of resiliently retained dentures is controversial. Limited data exist on the long-term survival of resiliently retained CDPs, with few retrospective studies and no randomized clinical trials comparing rigid and resilient bearings. In contrast, extensive studies are available for double-crown-retained RPDs (see Table 1). The aim of the present study was to assess the long-term survival rate and need for maintenance of resiliently retained CDPs. Although some studies have previously investigated this topic, most were based on comparatively limited datasets or shorter observation periods. In contrast, the present study analyses a uniquely large dataset on CDPs comprising extensive clinical information with longitudinal follow-up. To the best of our knowledge, no previous study has assembled and systematically evaluated such a comprehensive amount of time-based clinical data in this context. This approach allows a more robust assessment of the investigated factors and provides new insights on CDPs beyond those reported in the existing literature. 

**Table 1:** Survival rates of double-crown retained removable partial dentures

Author	Year	Type of denture	Survival rate
Bergman et al.	1996	conical crown retained removable partial dentures	78,3% after 73–92 months (dentures)
Igarashi et al.	1997	conical crown retained removable partial dentures	on average 13,7% abutment tooth loss in 10 years
Wenz et al.	1998	resilient crown retained removable partial dentures	87% after 5 years (abutment teeth)
			80% after 10 years (abutment teeth)
Wenz et al.	2001	resilient crown retained removable partial dentures	84% after 5 years (abutment teeth)
			66% after 10 years (abutment teeth)
Widbom et al.	2004	telescopic crown retained removable partial dentures	1,33% denture loss after 9 years
			7% abutment tooth loss after 9 years
Piwowarczyk et al.	2007	double crown retained removable partial dentures	6,7% abutment tooth loss after 5 years
Wöstmann et al.	2007	telescopic crown retained removable partial dentures	95,1% after 5 years (dentures)
			95,3% after 5 years (abutment teeth)
Schwindling et al.	2014	telescopic crown retained removable partial dentures	90% after 7 years (dentures)
		conical crown retained removable partial dentures	78,5% after 7 years (dentures)
		resilient crown retained removable partial dentures	78,5% after 7 years (dentures)
Ishida et al.	2017	double crown retained removable partial dentures	100% after 5 years (dentures)
			96,8% after 5 years (abutment teeth)
Rinke et al.	2019	resilient telescopic crowns	62% after 5 years (dentures) 38% after 8 years (dentures)
Yoshino et al.	2020	double crown retained removable partial dentures	94,7% after 10 years (dentures)
			83,8% after 10 years (abutment teeth)
			70,8% after 20 years (dentures)
			66,3% after 20 years (abutment teeth)
Prott et al.	2025	double crown retained removable partial dentures	96.6% after 5 years
			88.2% after 10 years
			61.7% after 20 years
			38.3% after 30 years

## Materials and methods

Ethical approval was obtained from the Ethics Committee of the Johann Wolfgang Goethe-University Frankfurt (103/19). Using patient records, all CDPs inserted between 2008 and 2018 were recorded, tracking follow-ups, modifications or repairs. CDPs still in situ at the end of the observation period were considered successful. A further distinction was made between 100% success (overall survival) and survival of the work (functional survival). Overall survival means all original abutment teeth were still in situ at the end of the examination period. Functional survival means one or more abutment teeth were lost, but the CDP still functions as such at the end of the observation period. Failures included total abutment loss, irreparable fractures, or non-wearability. A total of 299 CDPs with 619 abutment teeth were inserted in 267 patients at the Department of Prosthodontics at the ZZMK Carolinum of the Johann Wolfgang Goethe-University Frankfurt. The selection of patients included people of all age groups and sexes, who had been in recall for at least six months. Sufficient documentation of the findings before and during the wearing period of the CDP, an initial finding and at least one recall finding and documentation of the prosthesis- or abutment-related aftercare measures carried out were required. This resulted in a case number of 204 patients with a total of 227 CDPs and 466 abutment teeth.

The quantitative parameters recorded were examined descriptively. Dependences between parameters were tested using Pearson’s chi-square test, followed by log-rank testing. The critical (*P* < 0.05) and near-critical variables were further examined by Cox regression. The survival and success rates were determined using the Kaplan-Meier method. In addition, recurrent events were analysed using the Non-Parametric Mean Cumulative Function (MCF) and compared for the critical (*P* < 0.05) and near-critical parameters. The MCF indicates the average number of events per case that can be expected at a specific point in time. RStudio (Posit PBC [formerly RStudio Inc.]) and MATLAB (The MathWorks, Inc.) were used to perform statistical calculations.

## Results

In preparation for statistical calculations, the collected parameters were tested for independence using Pearson’s chi-square test. By definition, if there is a dependency between two parameters, they must not be evaluated together as two independent influencing factors when calculating a Cox regression. Non independent variable pairs according to the Pearson’s chi-square test were the localization of the prosthesis and the opposing jaw restoration, the number of abutment teeth and the distribution of abutment teeth as well as abutment tooth fracture and abutment tooth loss.

### Patients

The average patient age was 67 years. Of 204 patients, 43.6% were female and 56.4% were male. The mean follow-up duration was 41 months (3.4 years). The longest follow-up duration was 122 months (10.167 years), the shortest 6 months (0.5 years). Of 227 cases included, 41% attended a recall every six months, 32% attended annually and 28% attended less frequently than annually.

### Dentures and abutment teeth

Of 227 CDPs examined, 51% were fabricated in the maxilla and 49% in the mandible. 27,75% of 227 CDPs were supported on 1 abutment tooth, 40,97% on 2 abutment teeth and 31,28% on 3 abutment teeth. 41.4% of CDPs showed a punctual arrangement of the abutment teeth, 37,45% a linear distribution and 21,15% a triangular distribution. The Pearson’s chi-square test revealed a dependency between the distribution and the number of abutment teeth, indicating that a spatial distribution of the abutment teeth in the jaw is only possible if more than one tooth is present. Of 227 cases examined, fixed partial dentures of the opposing jaw were found in 11%, RPDs of the opposing jaw in 54% and a full denture in the opposing jaw in 35%.

The 227 CDPs examined were anchored on a total of 466 abutment teeth. The canines of all quadrants served as abutment teeth most frequently in relation to the other teeth, followed by maxillary anterior teeth and mandibular premolars. The primary crowns were made of a precious alloy in 60.4% of cases and of a non-precious alloy in 39.6% of cases. The abutment teeth were priorly restored with a crown in 70.5% of the cases, with a filling in 18.1% of the cases and with no restoration in 11.5% of the cases. 85.5% of abutment teeth were vital at the time of insertion of the CDP. 55.5% of the teeth had no degree of mobility when the CDP was incorporated, 36.6% of the teeth had a mobility of I and 7.9% of the teeth had a mobility of II.

### Changes in abutment teeth vitality and mobility

In 15.9% of cases, a loss of vitality occurred in one abutment tooth, in 3.5% cases in two abutment teeth, meaning they required endodontic treatment. The 183 cases in which no change in vitality occurred also include the 33 cases with initially avital abutment teeth, meaning they already were endodontically treated. Statistically significant for the change in abutment vitality are the patient age at insertion, the total duration of the examination and the initial abutment vitality (*P* < 0.05).

The change in abutment teeth mobility is defined as the difference in the records in the patient file between the time of insertion of the prosthesis and the last file entry. In 65.2% of cases, no change in tooth mobility was found, in 17.2% an increase and in 17.6% a decrease. The age at insertion and the total duration of the examination significantly influenced the change in tooth mobility (*P* < 0.05).

### Minor and major complications

The following parameters were classified as complications: decementation of a primary crown, abutment tooth fracture, abutment tooth loss and denture fracture. The loss of an abutment tooth is a complication that cannot be reversed. The other biological and technical complications can all be treated.

**Fig. 1:**
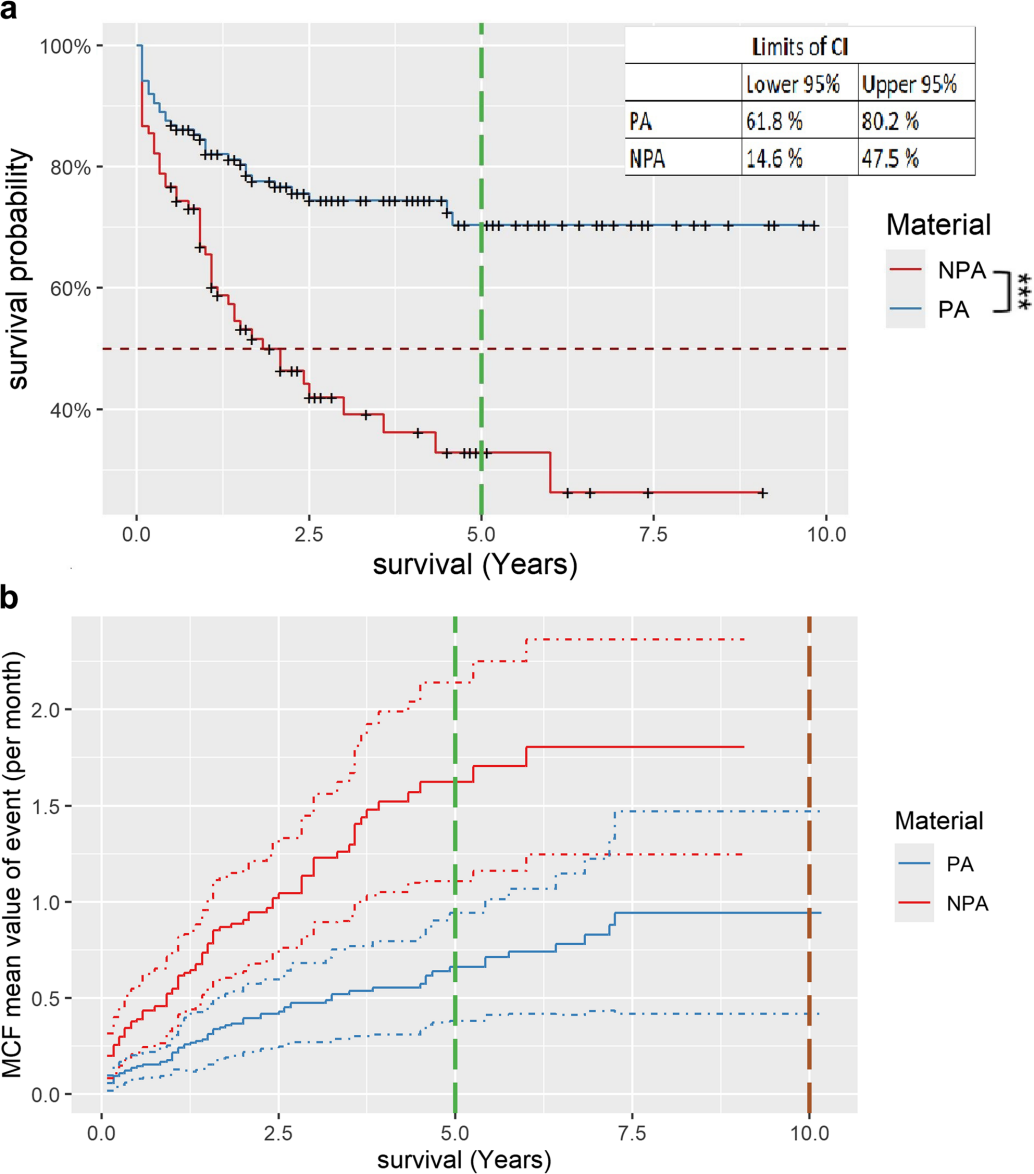
Kaplan-Meier survival curve of decementation and Mean Cumulative Function for recurring events subdivided by primary crown material. **a**, Blue curve shows the survival probability of primary crowns made of a precious alloy (PA), red curve shows the survival probability of primary crowns made of a non-precious alloy (NPA). The CI intervals for the last entry of the curve are given in the upper right corner. Statistically significant difference between the two is marked by asterisk [** = significance]. **b**, Blue curve shows the mean value of a decementation for primary crowns made of a precious alloy (PA), red curve shows the mean value of a decementation for primary crowns made of a non-precious alloy (NPA). Dotted lines represent the upper and lower CI of the curves. The vertical bars mark 5 and 10 years

In 63.4% of the CDPs examined no decementation occurred, in 46.7% decementation occurred one or more times. Related to the material of the primary crowns, 84.3% of the precious alloy primary crowns and only 68.3% of the non-precious alloy primary crowns showed no decementation. Statistically significant characteristics for decementation are the total examination duration, opposing jaw restoration, previous abutment restoration and material of primary crowns (*P* < 0.05). The Kaplan-Meier-curve (Fig. 1a) shows that the survival probability of crowns made of a gold alloy regarding the occurrence of decementation after 5 years is 70.4% [CI:61.8%; 80.2%], while that of non-precious alloy crowns is only 32.9% [CI:22.1%; 48.9%]. The difference between the two is statistically significant (*P* < 0.05). The MCF-curve in Fig. 1b confirms this statement. 5 years after the insertion of the CDPs, crowns made of a gold alloy were decemented less than 0.6 times per case on average while crowns made of non-precious alloy were decemented more than 1.5 times per case on average over the same period.

**Fig. 2:**
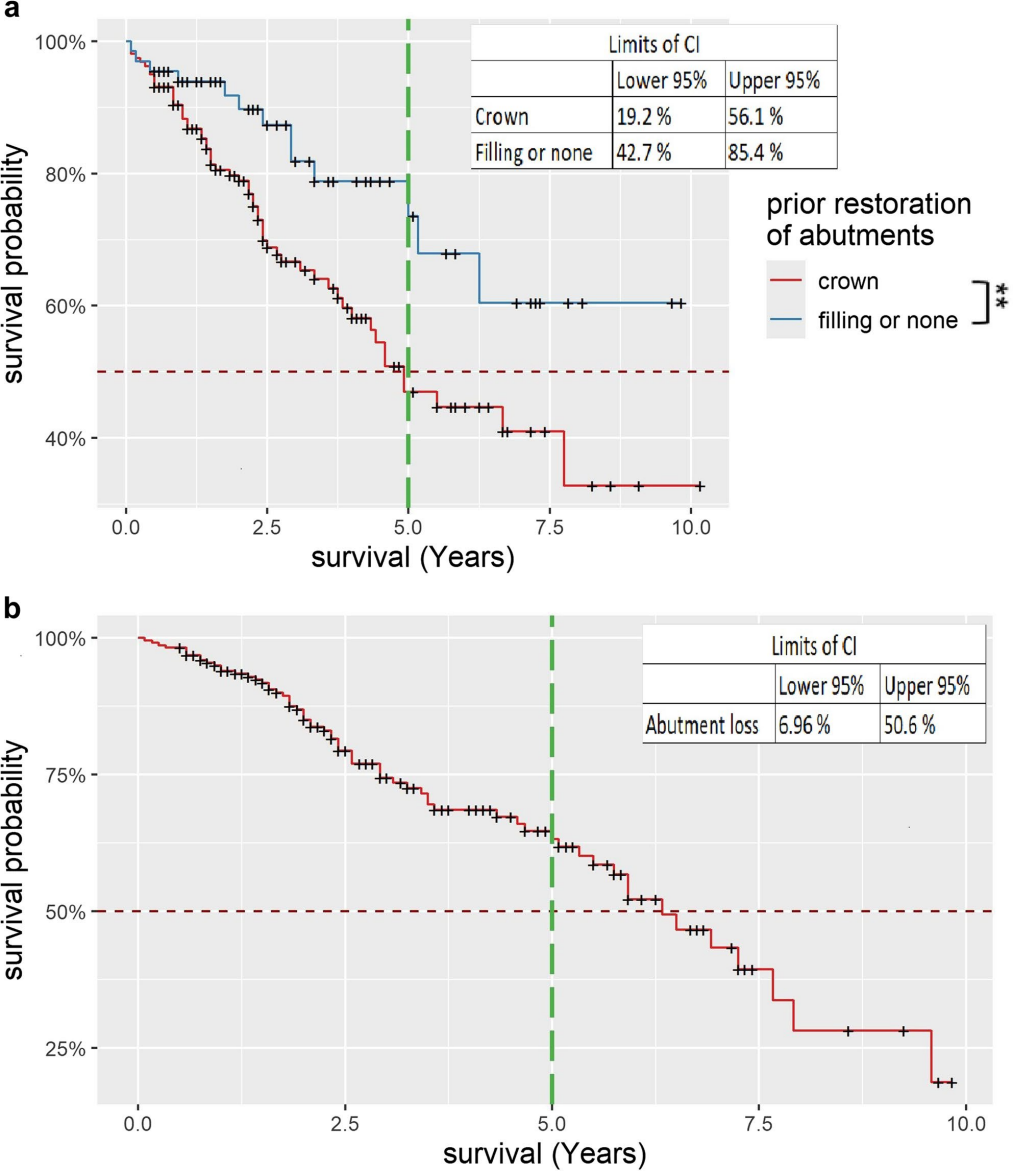
Kaplan-Meier survival curve abutment tooth fracture and abutment tooth loss. **a**, Abutment tooth fracture depending on previous restoration of the abutment teeth, red curve shows the survival probability for teeth priorly restored with a crown, blue curve shows the survival probability for teeth priorly restored with a filling or with no prior restoration. The CI intervals for the last entry of the curve are given in the upper right corner. Statistically significant difference is marked by asterisk [** = significance]. The vertical bar marks 5 years. **b**, Kaplan-Meier survival curve for abutment tooth loss. The CI intervals for the last entry of the curve are given in the upper right corner. The vertical bar marks 5 years

More than two thirds of cases (69.2%) remained free of an abutment tooth fracture over the entire observation period. If a fracture of a tooth occurred, then usually only once per case (22%). The previous abutment tooth restoration and the total duration of the examination had a significant influence (*P* < 0.05) on the occurrence of an abutment tooth fracture. Figure 2a shows the Kaplan-Meier survival curves for the abutment tooth fracture depending on the previous abutment restoration. It is noticeable that the survival curve for cases with abutment teeth previously restored with a crown (46.9% [CI:36.8%;59.8%] after 5 years) was worse than that for cases with previously unrestored teeth or teeth restored with a filling (73.6% [CI:59.9%;90.4%] after 5 years). The difference between the two is statistically significant (*P* < 0.05).

The loss of one or more abutment teeth occurred in a total of 28.7% of cases. It should be noted that in the case of the loss of one (17.6%) or two abutments (9.3%), no distinction was made as to whether a CDP had lost all its abutment teeth or whether one or two teeth remained. The loss of three abutment teeth (1.8%) means the complete loss of all abutment teeth of a CDP. Significant characteristics for abutment tooth loss (*P* < 0.05) are the age at insertion and total examination duration. Figure 2b shows the cumulative Kaplan-Meier survival curve for the occurrence of abutment tooth loss: after 5 years, all abutment teeth were still in situ in 63.2% [CI:55.02%;72.7%] of cases. The calculation of the MCF showed that on average 0.5 cases of abutment tooth loss had occurred after 5 years.

In 81.5% of cases, no denture fracture occurred, while in 18.5% it occurred one or more times. Significant characteristics for denture fracture (*P* < 0.05) are the age at insertion, the total duration of the examination and the opposing jaw restoration.

### Maintenance procedures

Follow-up maintenance procedures include pressure point relief in a CDP and relining of a CDP.

**Fig. 3:**
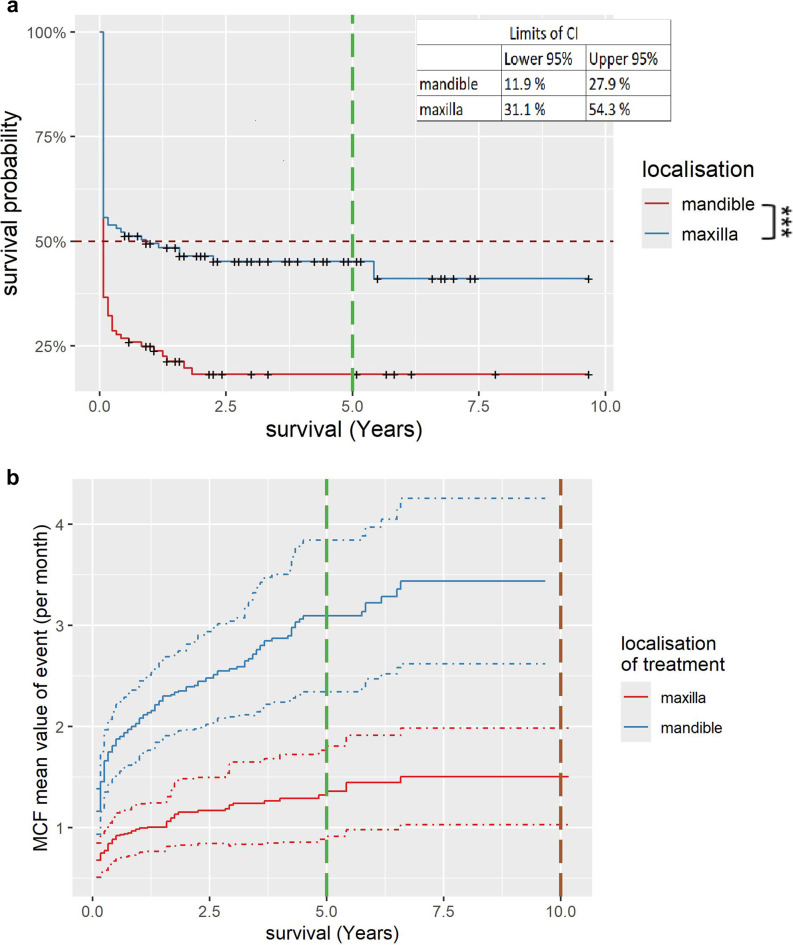
Kaplan-Meier survival curve for the occurrence of pressure points and Mean Cumulative Function for recurring events categorized by the localization of the restoration. **a**, Red curve = mandible, blue curve = maxilla. The CI intervals for the last entry of the curve are given in the upper right corner. Statistically significant difference marked by asterisk [*** = strong significance]. The vertical bar marks 5 years. **b**, Red curve = maxilla, blue curve = mandible. Dotted lines represent the upper and lower CI of the curves. The vertical bars mark 5 and 10 years

In 67% of cases, pressure points had to be relieved one or more times. Figure 3a shows the Kaplan-Meier survival curves for the occurrence of pressure points depending on the denture location. The survival curve shows that after 5 years, around 18.2% [CI:11.9%;27.9%] of the CDPs in the mandible had not experienced any pressure points, while the figure for the maxilla is around 45.2% [CI:36.8%;55.5%]. The difference between the two is statistically significant. The occurrence of pressure points is significantly dependent (*P* < 0.05) on the restoration of the opposing jaw and the localization of the restoration. If the localization of the restoration is considered, the average number of pressure points after 5 years was 1.3 times per case if the CDP was incorporated in the maxilla, but 3.2 times per case if the CDP was incorporated in the mandible (see Fig. 3b).

The CDP had to be relined at least once in 29.5% of cases, while 70.5% of cases did not require relining. The statistically significant characteristics for relining (*P* < 0.05) are the total examination duration and recall frequency.

### Success of CDP

A CDP still in situ at the end of the observation period was considered a success. Success was further subdivided into overall survival and functional survival. Overall survival means all original abutment teeth were still in situ at the end of the examination period and only minor complications occurred. Functional survival means one or more abutment teeth were lost, meaning a major complication occurred in addition to minor complications, but the CDP still functioned as such at the end of the observation period. All CDPs that were no longer in situ as CDPs at the end of the examination period were considered failures.

The following characteristics were identified as significant for success using the log-rank test and Cox-regression: number of abutment teeth (P:0.0002), loss of abutment teeth (P:0.0000), relining (P:0.002). The results of the Cox regression calculation for the three significant characteristics are showed in Table 2. The parameters defined above as complications do not directly influence the success of the restoration. However, they are intertwined with the parameters listed here. 74.9% of CDPs were considered a success, while 25.1% of CDPs were considered a failure. Table 3 shows the successes and failures of the CDPs in absolute figures.

**Table 2:** Cox regression of characteristics that significantly influence the success of CDPs

		Hazard Ratio	CI Upper 95%	CI Lower 95%	*P*-value
Success					
number of abutment teeth	1	0.152	0.742	0.031	0.0002
	2	0.143	0.729	0.028	
	3	0.723	0.965	0.542	
loss of abutment tooth	no	0.926	0.886	0.967	0.000
	yes	0.056	0.01	0.327	
relining	no	0.363	0.168	0.786	0.002
	yes	0.182	0.042	0.796	

The parameters number of abutment teeth, loss of abutment tooth and relining were defined as significant for the success of CDP. The table enumerates the hazard ratio, upper and lower CI intervals and *P*-values for each parameter

**Table 3:** Success or failure of CDPs

Success / failure	Number of abutment teeth			Distribution of abutment teeth		
	1 AT	2 AT	3 AT	punctual	linear	triangular
Failure	25	23	9	31	20	6
Success	38	70	62	63	65	42
total	63	93	71	94	85	48

Success or failure according to number of abutment teeth (AT) or abutment teeth distribution. In total, 170 cases are successful and 57 cases are a failure

88.2% of the prostheses were considered overall survival and 11.8% functional survival. The parameters defined as complications were not included in the calculation of functional and overall survival, as these are minor complications that can be remedied. Only abutment tooth loss counts as a major complication, as tooth extraction is irreversible, and was considered. The most common complication in successful CDPs was decementation of a primary crown in 20% of cases, in a further 19% of cases decementation was accompanied by abutment tooth fracture, abutment tooth loss or both. Almost half of all successful CDPs (41.8%) were complication-free during the entire observation period. The aftercare of all successful cases consisted of pressure point relief in 46.5% of cases, relining in 3.5% of cases and both in 18,8% of cases.

Total abutment tooth loss and non-wearable prosthesis were defined as reasons leading to failure of the restoration. The Pearson’s chi-square test showed that abutment tooth loss and abutment tooth fracture are very closely related (*P*:0.0002), i.e. an abutment tooth loss is almost always precedented by abutment tooth fracture. 73.7% of CDPs that were considered failures lost all abutment teeth. 35.1% of the failures due to abutment tooth loss were dentures borne on one abutment tooth, 31.6% were dentures borne on two abutment teeth and 7% were dentures with three abutment teeth. 26.3% CDPs classified as failures had to be labeled as such because the prosthesis could not be worn by the patient.

### Survival of CDP

**Fig. 4:**
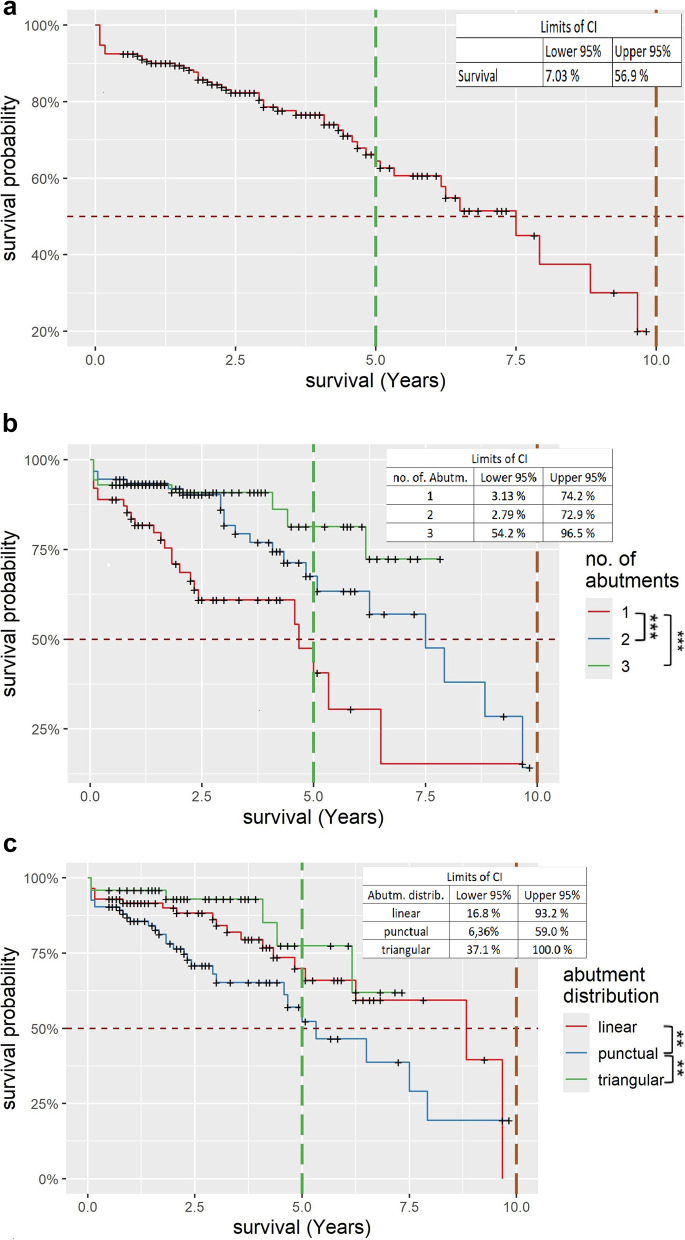
Survival rates of Cover-Denture prostheses. **a**, Cumulative Kaplan-Meier survival rate of all examined Cover-Denture prostheses. The CI intervals for the last entry of the curve are given in the upper right corner. The vertical bars mark 5 and 10 years. **b**, Survival probability according to Kaplan-Meier categorized by abutment teeth number. The CI intervals for the last entry of the curve are given in the upper right corner. Statistically significant differences marked by asterisks [*** = strong significance]. The vertical bars mark 5 and 10 years. **c**, Survival probability according to Kaplan-Meier categorized by abutment distribution. The CI intervals for the last entry of the curve are given in the upper right corner. Statistically significant differences marked by asterisks [** = significance]. The vertical bars mark 5 and 10 years

The Kaplan-Meier survival rate 3 years after insertion of the CDPs was 78.6% [CI:72.48%;85.3%], and 5 years after insertion it was 64.5% [CI:55.48%;74.9%] (Fig. 4a). 50% were reached after 7.4 years (89 months). After 10 years, the survival probability was approximately 20.0% [CI:7.03%;56.9%]. CDPs with a single abutment tooth had a 3-year survival rate of 61.0% [CI:48.5%;76.6%] and a 5-year survival rate of 40.6% [CI:24.27%;68.1%]. CDPs anchored on two abutment teeth had a 3-year survival rate of 81.7% [CI:72.3%;92.3%] and a 5-year survival rate of 67.6% [CI:54.77%;83.4%]. CDPs anchored on three abutment teeth had a 3-year survival rate of 90.9% [CI:84.2%;98.2%] and a 5-year survival rate of 81.4% [CI:68.5%;96.7%] (Fig. 4b). The differences in the survival probability of one and two as well as one and three abutment teeth are statistically significant.

When comparing the survival probability curves subdivided by the number of abutment teeth (Fig. 4b) and by abutment teeth distribution (Fig. 4c), it can be observed that CDPs anchored on a single abutment tooth exhibited increased failure rates in the first three years, stabilizing thereafter. CDPs with multiple abutment teeth exhibited a similar behaviour up to 34 months after insertion, still, after 3 years, the survival probability of prostheses on multiple abutment teeth started to drift apart, those anchored on two abutments dropped to 81.7% [CI:72.3%;92.3%] compared to those on three abutments at 90.9% [CI:84.2%;98.2%]. The curves in Fig. 4c, on the other hand, are not so far apart. After 5 years, the survival probability for CDPs with a triangular abutment teeth distribution was 77.4% [CI:59.3%;100%], with a linear abutment teeth distribution 69.8% [CI:57.2%;85.2%] and with a punctual abutment teeth distribution 52.3% [CI:38.14%;71.7%]. The differences in the survival probability between linear and punctual abutment teeth distribution as well as between punctual and triangular distribution are statistically significant.

Vertical lines have been added to the graphs to make it easier to read the survival rates. These mark the time points 5 and 10 years.

## Discussion

The present study is one of few studies investigating the survival rates of resiliently retained RPDs. It highlights significant findings on the survival and complications of CDPs, aligning with existing literature while addressing factors such as abutment restoration and material influences. For the first time, recurrent events were examined using the Non-Parametric Mean Cumulative Function (MCF). The present study is based on a large clinical dataset on CDPs with longitudinal follow-up. The substantial amount of time-based clinical information allowed not only the assessment of overall survival, but also a separate analysis of functional survival, thereby providing additional insight into the clinical course of these prostheses. Moreover, the documentation of follow-up visits made it possible to systematically evaluate maintenance measures associated with CDPs. This combination of a large dataset, longitudinal clinical information, and the differentiated analysis of survival outcomes provides a broader and more robust understanding of CDP performance than has been reported in previous studies.

However, the study has limitations due to its retrospective design, relying solely on patient records. Of 298 patients receiving a CDP at the Department of Prosthodontics at the ZZMK Carolinum of the Johann Wolfgang Goethe-University Frankfurt between 2008 and 2018, 204 met inclusion criteria (68.5%). Follow-up rates in other studies vary: Rehmann et al. [21] and Rinke et al. [22] documented higher rates, while Schwindling et al. [23] reported a significantly lower rate.

Statistical analysis was conducted per CDP. The Kaplan-Meier method [24, 25] calculated survival rates, while MCF estimates provided insights into recurring complications and follow-up measures. The MCF estimator is a tool primarily used in engineering sciences to analyse recurring events. Unlike Kaplan-Meier survival analyses, which censor a case after the first occurrence of an event, it can map recurring events, making it suitable in this case for mapping multiple complications or follow-up measures and making accurate statements about the maintenance needs of CDPs. Recurring complications and follow-up measures in CDPs have not yet been depicted in this form in the relevant literature, so the present study offers added value for daily practice. To determine which of the recorded parameters significantly influence the success of CDPs, log-rank tests were performed and parameters were further analysed using Cox regressions in a forward-planning design. This represents a classic approach to the analysis of time-dependent variables [26].

The study included younger and older patients, with older individuals potentially facing memory impairments or motor skill limitations affecting oral hygiene, influencing periodontal parameters and carious lesions [11, 16].

A total of 227 CDPs on 466 abutment teeth in 204 patients were examined, comparable to other studies [22, 27–31]. Sex distribution and average patient ages align with other studies [4, 23, 27, 31]. Age significantly influenced abutment vitality, tooth mobility, and denture fracture, while sex did not. Unlike others, age did not significantly influence success of CDPs [32]. Follow-up averaged 41 months (3.4 years), ranging from 6 to 122 months. Wenz et al. [33] reported similar durations, while Rinke et al. [22] noted longer follow-ups. The average follow-up duration is comparable with other studies [4, 21, 22, 28–30, 33, 34]. The total examination duration significantly influenced decementation, change in abutment vitality, change in tooth mobility, abutment fracture and loss and denture fracture and relining. A longer follow-up period increases the risk of complications or aftercare measures occurring, partly because aging patients experience motor skills limitations.

CDPs were limited to three or less abutment teeth with resilient telescopic crowns. Comparisons with studies using different numbers of abutments or anchoring methods indicate similar findings. Thus, the results found here are comparable with the relevant literature, even though no separate survival analysis of the abutment teeth was carried out in this study. The number of abutments significantly influenced the success of the dentures, which is in line with other publications [32].

Comparisons of the present study with others [20, 21, 30] regarding abutment tooth vitality show similar rates. Periodontal changes (i.e. changes in abutment tooth mobility), possibly due to hygiene [35], denture fit [12–14, 16] or physiological aging processes [36] were observed, but remained stable in most cases. Still, there are studies suggesting that RPDs increase the risk of tooth loss in periodontally damaged dentitions [37].

All abutment teeth of one CDP were restored with primary crowns made of the same alloy. Primary crowns made of precious alloy were less prone to decementation and had better survival rates. In Rinke et al. [22], as well as in studies by Behr et al. [38], Widbom et al. [4] and Wöstmann et al. [5], all primary and secondary parts were made of precious alloys. These findings highlight the preference for precious alloys in double-crown retained RPDs, likely due to their favorable clinical performance and material properties [39]. This is consistent with the significant influence of the material of the primary crowns on the decementation of a primary crown.

Unlike most studies on double-crown retained RPDs, this study also examined prior restoration of the abutment teeth. Prior restoration significantly impacted decementation, abutment tooth fractures, and nearly significantly affected abutment tooth vitality. The influence of prior restoration on the parameters listed here may be due to reduced tooth structure and previous traumatic influences (e.g. caries or drilling trauma), which can ultimately lead to vitality loss.

In the present study, 33% of CDPs remained free of complications over the observation period. Behr et al. [38] state a similar value of 34.2% for telescopic prostheses.

Decementation of a primary crown was significantly influenced by crown material, prior restorations, opposing jaw restoration, and follow-up duration. Precious alloy crowns performed better than non-precious alloys. Various authors found decementation rates between 20.6% and 76.8% [5, 23, 30, 38]. This shows that the decementation rate of 36.6% found in the present study is in line with the results of other authors.

The occurrence of an abutment fracture did not depend on the number or distribution of the abutment teeth. However, the previous abutment tooth restoration and the total duration of the examination had a significant influence on this event. The Kaplan-Meier analysis for all cases showed a survival rate of 54.8% after 5 years regarding the occurrence of an abutment tooth fracture. Widbom et al. [4] indicate a cumulative probability of 22% for fracture of an abutment tooth and decementation of a primary crown. In a meta-analysis [15], the prevalence of abutment tooth fracture is given as 1-1.7% of cases. The result of the present study clearly exceeds the values listed in the literature. Due to the integration of teeth with a reduced prognosis into the CDPs, it can still be considered sufficiently good that more than half of the cases have not experienced an abutment tooth fracture after 5 years.

Abutment tooth loss was observed in 29.6% of cases, with a 5-year survival rate of 63.2%. Fractures often preceded losses, as the Pearson’s chi-square test revealed a dependency between abutment tooth loss and abutment tooth fracture (*P*:0.0002). Despite higher loss rates compared to other studies [4, 20, 21, 33, 40, 41], the results are acceptable considering the inclusion of teeth with reduced prognosis. Age significantly influenced abutment tooth loss, a result equally found by others [32, 37].

Pressure points were relieved more frequently in mandibular CDPs and for fixed opposing jaw restorations. One explanation for this lies in the twisting of the mandibular brace when the lower jaw moves and in the fact that the lower jaw offers less support for a mucogingival-supported prosthesis than the upper jaw. The reduced support of the prosthesis and possible lateral deflection movements in the lower jaw favor the development of pressure points. A fixed opposing denture in occlusion may exert higher forces on the mucosa-supported CDP, which is why it becomes more embedded in the mucosa and causes pressure points. Pommer et al. [42] also state in a review that the fabrication of a RPD is more suitable for patients with an opposing jaw that is already completely edentulous and fitted with a complete denture. This is in contradiction to a study by Prott et al., which determined a complete denture in the opposing jaw as a key risk factor for loss of double-crown retained RPDs [32].

The number of abutment teeth, abutment tooth loss and relining had significant influence on CDP success. CDPs with fewer abutment teeth failed more often due to complete abutment tooth loss, with longer survival for those anchored on three teeth. Prott et al. determined three or less abutment teeth as a key risk factor for loss of double-crown retained RPDs [32]. Still, unlike Rinke et al. [22] the present study found more successes with two abutment teeth. Long-term success depends on preserving supporting tissues through force distribution, which becomes challenging with few teeth. Hofmann’s resilient anchorage principle [17, 18] addresses this by reducing load on remaining teeth. The 5-year survival rate (64,5%) aligns with Rinke et al. [22], but this study’s complication-free success rate (41.8%) exceeds their 13%. Wenz et al. reported 84-93.8% abutment survival at 5 years and 66-89.4% at 10 years [20, 33], while Schwindling et al. [23] found 78.5% survival at 7 years. Compared to a 1-year survival rate of 85.7% for overdentures borne on 2 implants reported by Onclin et al. [3], the survival rate found in this study can be rated as good. This study’s 3-year (78.6%) and 5-year (64.5%) survival rates are lower than other studies on resilient anchorage but fall within the lower mid-range of other studies, which report 50%-100% survival at 5–6 years [5, 35, 43], 39.5%-90% at 7–8 years [23], 80.2%-94.7% at 10 years and 51%-70.8% at 20 years [27, 32], and 27% after 30 years [32]. Survival rates for double-crown RPDs are heterogeneous. The 5-year survival rate determined in the present study is in the lower mid-range of the studies mentioned here, with this study focusing solely on resiliently anchored prostheses compared to rigidly anchored ones in other research. A prospective study, in which a defined recall interval for periodontal follow-up is adhered to, can provide more precise data regarding the survival and success rate of overdenture prostheses and their abutment teeth.

## Conclusion

Based on the present results, CDPs represent a suitable alternative to complete extraction and can be recommended in clinical practice, especially when implants are not an option because of financial or morphological issues. Survival rates differ based on the number of abutment teeth incorporated in the denture. CDPs anchored on three or two abutment teeth, and CDPs with a triangular abutment teeth distribution, show better survival rates than CDPs anchored on one abutment tooth or with linear or punctual abutment teeth distribution. Thus, the more remaining teeth can be incorporated as abutment teeth, the better the prognosis. Still, the determined survival rates can be considered acceptable, measured against the assumption that a CDP is intended to facilitate the transition to a full denture for the patient. The teeth integrated as abutments in the construction are protected by the primary crowns and remain accessible for oral hygiene measures since the superstructure is removable. A weakness appears to be abutment tooth fracture and subsequent abutment tooth loss, with a frequently occurring complication being the decementation of primary crowns. Due to a lower decementation rate, it can be recommended to prefer the fabrication of primary components from a precious alloy. Other complications or follow-up measures do not occur more frequently than with other types of RPDs.

## Data Availability

No datasets were generated or analysed during the current study.
